# Gaps and barriers in health-care provision for co-morbid diabetes and chronic kidney disease: a cross-sectional study

**DOI:** 10.1186/s12882-017-0493-x

**Published:** 2017-02-28

**Authors:** C. Lo, H. Teede, G. Fulcher, M. Gallagher, P. G. Kerr, S. Ranasinha, G. Russell, R. Walker, S. Zoungas

**Affiliations:** 1 Diabetes and Vascular Research Program, Monash Centre for Health Research and Implementation, School of Public Health and Preventive Medicine, Monash University, Clayton, Victoria Australia; 2 Diabetes and Vascular Medicine Unit, Monash Health, Clayton, Victoria Australia; 3 Department of Diabetes and Endocrinology, Royal North Shore Hospital, St Leonards, New South Wales Australia; 4 Department of Nephrology, Concord Hospital, Concord, New South Wales Australia; 5 The George Institute for Global Health, Sydney, New South Wales Australia; 6 Department of Nephrology, Monash Health, Clayton, Victoria Australia; 7 School of Primary Health Care, Monash University, Notting Hill, Victoria Australia; 8 Department of Renal Medicine, Alfred Health, Prahran, Victoria Australia

**Keywords:** Diabetes, Chronic kidney disease, Multi-morbidity, Health-care, Tertiary health-care, Treatment gaps, Barriers

## Abstract

**Background:**

Patients with diabetes and chronic kidney disease (CKD) are a complex subset of the growing number of patients with diabetes, due to multi-morbidity. Gaps between recommended and received care for diabetes and chronic kidney disease (CKD) are evident despite promulgation of guidelines. Here, we document gaps in tertiary health-care, and the commonest patient-reported barriers to health-care, before exploring the association between these gaps and barriers.

**Methods:**

This cross-sectional study recruited patients with diabetes and CKD (eGFR < 60 mL/min/1.73 m^2^) across 4 large hospitals. For each patient, questionnaires were completed examining clinical data, recommended care, and patient-reported barriers limiting health-care. Descriptive statistics, subgroup analyses by CKD stage and hospital, and analyses examining the relationship between health-care gaps and barriers were performed.

**Results:**

308 patients, of mean age 66.9 (SD 11.0) years, and mostly male (69.5%) and having type 2 diabetes (88.0%), participated. 49.1% had stage 3, 24.7% stage 4 and 26.3% stage 5 CKD. Gaps between recommended versus received care were evident: 31.9% of patients had an HbA1c ≥ 8%, and 39.3% had a measured blood pressure ≥ 140/90 mmHg. The commonest barriers were poor continuity of care (49.3%), inadequate understanding/education about CKD (43.5%), and feeling unwell (42.6%). However, barriers associated with a failure to receive items of recommended care were inadequate support from family and friends, conflicting advice from and poor communication amongst specialists, the effect of co-morbidities on self-management and feeling unmotivated (all *p* < 0.05).

**Conclusions:**

Barriers to health-care varied across CKD stages and hospitals. Barriers associated with a deviation from recommended care were different for different items of care, suggesting that specific interventions targeting each item of care are required.

**Electronic supplementary material:**

The online version of this article (doi:10.1186/s12882-017-0493-x) contains supplementary material, which is available to authorized users.

## Background

Diabetes is increasing in incidence and prevalence globally, with 8.3% of adults estimated to be affected [1]. Diabetes commonly co-exists with chronic kidney disease and accounts for up to 50% of people who develop end-stage kidney disease (ESKD) [2].

Together, co-morbid diabetes and CKD is an exemplar of the global health challenge from multi-morbidity, defined as the coexistence of 2 or more chronic conditions where 1 is not necessarily more central than the other [3]. Multi-morbidity is increasing as the population ages and the rate of non-communicable diseases increases [4] and is associated with poorer quality of life, and higher mortality rates [3]. The challenges of multi-morbidity are not well addressed by contemporary health care systems or clinical research, which have been framed around single chronic diseases [3, 4].

The co-existence of diabetes and CKD has an additive effect to increase the risk of cardiovascular disease [5], with a substantial increase in risk of premature mortality as renal function deteriorates towards end-stage kidney disease [2]. Health-care costs are also significant with the estimated unadjusted total expenditure for people with co-morbid diabetes and CKD in the USA being more than US $43 billion even in 2011 [6].

Despite the recent publication of guidelines and consensus statements for the management of co-morbid diabetes and CKD [7–10], there is emerging evidence that the care of patients with these co-morbidities is suboptimal. Studies report a gap between recommended care, as suggested by guidelines, versus received care − a significant proportion of patients fail to meet treatment targets, and other recommended health indicators of quality clinical care such as treatment of cardiovascular risk factors or anaemia [11–14]. Additionally, there is a paucity of knowledge surrounding the underpinnings of this gap and the relative importance of clinical and health system barriers to health-care, specifically for patients with co-morbid diabetes and CKD. This is a necessary step towards improving medical care and finding an effective and efficient health-care delivery system for this high risk population [7].

The objectives of this study were to 1) identify the degree to which patients with co-morbid diabetes and CKD experience recommended health-care as per internationally endorsed guidelines in the setting of tertiary health-care 2) quantitatively establish the most significant barriers to health-care, from the perspective of patients in the setting of tertiary health-care and 3) examine the association between failure to receive guideline recommended health-care (care gaps) and the patient-reported barriers to health-care.

## Methods

### Study design and setting

This Australian multi-centre study was a research collaboration between tertiary hospitals, research institutes, national consumer stakeholder groups (Diabetes Australia and Kidney Health Australia) and primary care groups. The study was conducted across ambulatory diabetes and renal clinics (serviced or supervised by diabetes and kidney specialists) of 4 large tertiary hospitals in Australia’s 2 most populous cities (Alfred and Monash Health in Melbourne and the Royal North Shore and Concord Hospitals in Sydney) from January to September 2014. These specialist ambulatory services communicate with primary care via written correspondence or phone calls but without any further integration of health-care such as shared medical records or care plans. Additionally, the ambulatory services of 2 of the hospitals (2 and 4), were structured such that the patient saw the same clinician each visit. Methods and results are presented in accordance with the STROBE (Strengthening The Reporting of Observational Studies in Epidemiology) guidelines [15]. The study was approved by the Monash University Human Research Ethics Committee (HREC) and the HREC of all participating sites.

### Participants

Patients with both diabetes and CKD were recruited from either ambulatory diabetes or renal clinics of each participating tertiary hospital over a 3-month period between January to September 2014. Patients were defined as having diabetes if the diagnosis was noted on medical records and/or confirmed by laboratory results as per World Health Organisation (WHO) criteria [16]. Patients were defined as having CKD if they had a sustained estimated glomerular filtration rate (eGFR) < 60 mL/min/1.73 m^2^ calculated using the CKD-EPI (Chronic Kidney Disease Epidemiology Collaboration) equation [17] (i.e. 2 or more eGFR readings) over a 3 month period. Pregnancy was an exclusion criteria. All participants gave written informed consent.

### Study conduct and variables

Each recruited subject participated in a survey consisting of two questionnaires. The first questionnaire was prospectively completed by the patient’s doctor during the clinic or from the doctor’s notes and laboratory results from clinic and collected demographic information (age, country of birth, language spoken at home) and other clinical characteristics (Additional file 1: Appendix: Part A). The second questionnaire was completed by the patient and examined patient-reported barriers to health-care (Additional file 1: Appendix: Part B) identified from the content analysis of 12 focus groups of 58 participants with co-morbid diabetes and CKD and 8 semi-structured interviews of carers from a previous multi-centre qualitative study performed by the authors [18] (Additional file 1: Appendix: Part A).

### Treatment targets for recommended care

The received care of participants was compared with treatment targets for recommended care from international guidelines [7–9, 19, 20]. The authors acknowledge the importance of individual application of recommended guideline care. For the purposes of this study, where more than one treatment target was offered for a particular parameter by different guidelines, the more conservative and simpler target was used. For example for HbA1c, the American Diabetes Association suggestion of HbA1c < 8% (64 mmol/mol) [7] was used rather than that from the European Renal Best Practice guidelines which varied from 7% (53 mmol/mol) to 9% (75 mmol/mol), depending on the patient’s clinical situation [8], or the KDOQI (Kidney Disease Outcomes Quality Initiative) guidelines which suggest a HbA1c of ~7% (53 mmol/mol) [9].

### Statistical analysis

Continuous variables were reported as means and standard deviations or medians with interquartile ranges if distributions were skewed. Categorical variables (including barriers), cardiovascular risk factors (e.g. hypercholesterolemia, hypertension, smoking etc.) and adherence to best practice guidelines were reported as frequencies and percentages. Likert scales were collapsed into 2 categories (disagree and agree) for questions related to barriers. Clinical characteristics were reported for the entire cohort and also according to KDOQI CKD stages. Characteristics between KDOQI CKD stages were compared using Chi square and Fisher Exact tests for categorical variables. For continuous variables, Student’s t tests for normally distributed and Kruskal-Wallis tests for skewed outcomes were used. Adjustments were made for clustering by hospital where possible.

Patient-reported barriers to health-care were ranked quantitatively according to percentages. Subgroup analyses for patients occurred according to KDOQI CKD stages (3, 4 and 5ND and 5D) [21] as it was felt that the health experiences of patients with KDIGO (Kidney Disease Improving Global Outcomes) CKD stage 3a and 3b would be similar. Chi square and Fisher Exact tests were used to compare between KDOQI CKD stages for recommended versus received care, adjusted for clustering by hospital. A linear trend analysis was also performed. An additional sub-analysis of patient-reported barriers to health-care was done by individual hospital using Chi square and Fisher Exact tests, with a Bonferroni correction.

The association between gaps in care (compared to recommended guideline care) and the patient-reported barriers to health-care were examined by comparing the percentage of patients who reported a barrier among those who received recommended care compared to those who did not, using Chi Square and Fisher Exact Tests, adjusted for clustering.

To evaluate the likelihood of a responder bias, the following variables were compared between responders and non-responders at one tertiary health service – age, gender, and KDOQI stages using the Student’s *t* test and Chi square statistic. A *p* value < 0.05 was considered significant except for when the Bonferroni correction was used when a *p* < 0.0125 was considered significant. All statistical analyses were performed using STATA v12.1.

## Results

### Participant characteristics

**Fig. 1:**
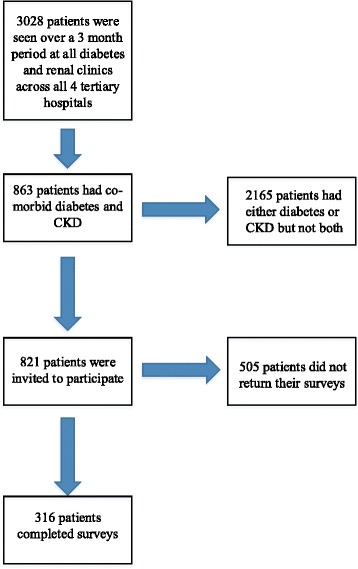
Patient recruitment

A total of 821 patients were invited to participate (see Fig. 1: Patient recruitment) with 316 patients completing surveys (response rate of 38.5%). Eight patients were excluded because their eGFR was ≥ 60 mL/min/1.73 m^2^ leaving 308 for the analysis. Patients (at one tertiary health service) who participated in the study were similar in age, gender distribution and KDOQI CKD stage to those who did not (Additional file 1: Appendix: Table S1).

Clinical characteristics, cardiovascular risk factors and biochemical parameters are reported in Table 1. The mean (SD) age of patients was 66.9 (11.0) years, 69.5% were male. The cohort was multi-cultural with less than half born in Australia (46.4%) and only 78% speaking English as the main language at home. The patients were generally overweight (mean body mass index or BMI 30.7; SD 7.4) and 94.5% had a history of hypertension. The majority of patients had type 2 diabetes (88.0%) of long duration (median 17, IQR 10–23.5 years). Patients were evenly distributed between KDIGO stages. Close to one-fifth of patients (19.2%) were receiving dialysis. The cohort had high cardiovascular risk with 47.0% having a history of ischemic heart disease. As expected and consistent with the pathophysiology and prognosis of later stage CKD, patients with earlier CKD stages tended to be slightly older, have a higher BMI, have a higher haemoglobin and HbA1c, have a lower phosphate and PTH, and have a lesser need for treatment for CKD metabolic bone disease, compared to later CKD Stages (Additional file 1: Appendix: Table S2: Characteristics and medication usage of patients with diabetes and CKD stratified by CKD stage).

**Table 1:** Characteristics and Medication usage of patients with diabetes and CKD

Clinical Characteristic	Total (*n* = 308)
Age (SD)	66.9 (11.0)
Male/Female (%)	214 (69.5)/94 (30.5)
Main language spoken at home (% English)	238/305 (78.0)
Australia as the country of birth	141/304 (46.4)
Body weight (IQR) kg	86.1 (72.8 – 101.3)
Height (SD) m	1.68 (0.10)
BMI (SD) kg/m^2^	30.7 (7.4)
Systolic blood pressure (SD) mmHg	134 (18)
Diastolic blood pressure (SD) mmHg	72 (11)
Active Smoker (%)	18/230 (7.8)
Diabetes duration (IQR) yrs.	17.0 (10.0 – 23.5)
Diabetes type (%)	
type 1	28 (9.1)
type 2	271 (88.0)
other	9 (2.9)
CKD stage (KDIGO)%	
3a	72 (23.4)
3b	79 (25.7)
4	76 (24.7)
5 (inclusive of 5D)	81 (26.3)
Dialysis (%)	59 (19.2)
Haemodialysis (%)	42 (71.1)
Peritoneal dialysis (%)	17 (28.9)
Other multi-morbidity	
Hypertension (%)	291/308 (94.5)
Dyslipidaemia (%)	256/308 (83.1)
Ischemic Heart Disease (%)	143/304 (47.0)
Stroke (%)	38/305 (12.5)
Peripheral Vascular Disease (%)	82/304 (27.0)
Diabetic Retinopathy (%)	132/305 (43.3)
Diabetic Neuropathy (%)	108/305 (35.4)
Diabetic Nephropathy as a cause of CKD (%)	219/306 (71.6)
Biochemical Parameters	
Haemoglobin (SD) g/L	122 (18)
HbA1c %	7.3 (6.4 – 8.3)
HbA1c (mmol/mol)	56 (47 – 67)
Total Cholesterol (IQR) mmol/L	3.9 (3.4 – 4.6)
LDL (IQR) mmol/L	1.8 (1.4 – 2.5)
HDL (IQR) mmol/L	1 (0.8 – 1.2)
Triglycerides (IQR) mmol/L	1.8 (1.2 – 2.5)
Potassium (SD) mmol/L	4.6 (0.6)
Calcium (SD) mmol/L	2.31 (0.14)
Phosphate (IQR) mmol/L	1.23 (1.08 – 1.46)
PTH (IQR) pmol/L	15.7 (7.8 – 30.6)
Medication Usage	
Insulin only (%)	136 (44.2)
Non-insulin glucose lowering therapy only (%)	85 (27.6)
Both insulin and non-insulin glucose lowering therapy (%)	67 (21.8)
Diet only with other glucose lowering therapies (%)	20 (6.5)
Statin use (%)	248 (80.5)
Fibrate (%)	34 (11.0)
Use of any antihypertensive (%)	278 (90.3)
Use of ACEI and/or AT2RB (%)	185 (60.1)
Use of other antihypertensive besides an ACEI/AT2RB (%)	240 (77.9)
ESA use (%)	64 (20.8)
Iron supplementation (%)	57 (18.5)
Phosphate binder (%)	58 (18.8)

### Recommended versus received care for co-morbid diabetes and CKD

Documented treatment targets and received care of patients as compared to recommended treatment targets and care according to guidelines and consensus statements [7, 8] are shown in Table 2. 31.9% of patients had an HbA1c ≥ 8% (64 mmol/mol), 39.3% of patients had a measured systolic blood pressure ≥ 140 mmHg or a diastolic blood pressure ≥ 90 mmHg, and 17.7% of those patients not on dialysis were not taking a statin. There were also screening gaps with 12.2% of patients with documented diabetic retinopathy not having an eye review over the preceding year; and 50.9% of patients with peripheral neuropathy not having their feet examined at their 3 monthly review.

**Table 2:** Guideline recommended care vs. received care

Recommended care	Met	Unmet	*P*
HbA1c < 8% (64 mmol/mol) when eGFR < 60 mL/min/1.73 m^2^ [7]	68.1% (192/282)	31.9% (90/282)	<0.00001
Blood pressure < 140/90 mmHg [7, 8]	60.7% (187/308)	39.3% (121/308)	<0.00001
A statin is recommended in patients with non-dialysis dependent CKD [7]	82.3% (205/249)	17.7% (44/249)	<0.00001
In the setting of diabetic retinopathy, eye examinations should be repeated annually by an optometrist/ophthalmologist [20]	87.8% (108/123)	12.2% (15/123)	<0.00001
In the setting of peripheral neuropathy feet should be examined every 3 months [20, 37]	49.1% (53/108)	50.9% (55/108)	0.68
Metformin should be ceased if eGFR ≤ 30 ml/min/m^2^ [7]	96.2% (154/160)	3.8% (6/160)	<0.00001
ESA^a^ prescribed if Hb < 100 g/L, allowing for individualisation [19]	50.0% (11/22)	50.0% (11/22)	1.00
Hb between 100 and 115 g/L while on ESA [19]	31.3% (20/64)	68.8% (44/64)	<0.00001

^a^ESA = Erythropoietin Stimulating Agent

**Fig. 2:**
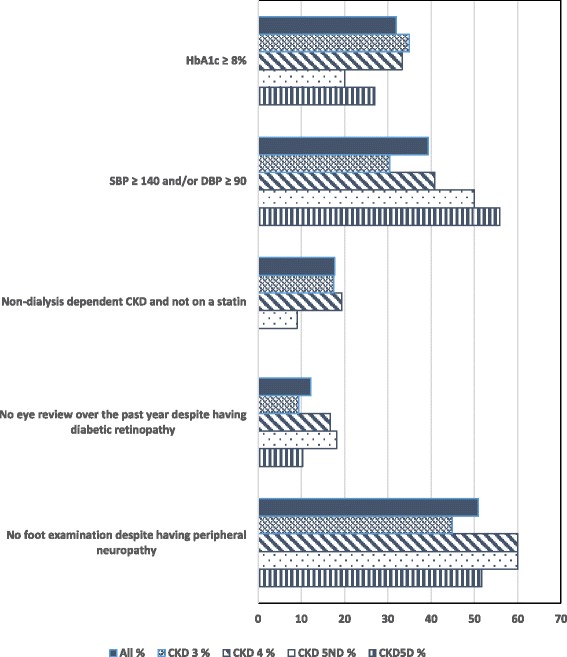
Deviation from optimal care according to CKD stage.  For all items of recommended care according to guidelines, comparisons between CKD stages were not statistically significant at the 5% level for Chi square or Fisher exact tests and for linear trends

On analysis by CKD stage i.e. CKD 3, 4, 5ND and 5D, although there were numerical differences between stages, there were no statistical differences in the proportion of patients who did not achieve recommended treatment targets including an HbA1c ≥ 8%, systolic BP levels ≥140 and/or diastolic BP levels ≥90 mmHg, not being on a statin for those with non-dialysis dependent CKD, not having an eye review over the past year despite the presence of diabetic retinopathy or not having a foot examination despite the presence of peripheral neuropathy (all *p* > 0.05, see Fig. 2).

### Barriers to health-care for co-morbid diabetes and CKD

**Fig. 3:**
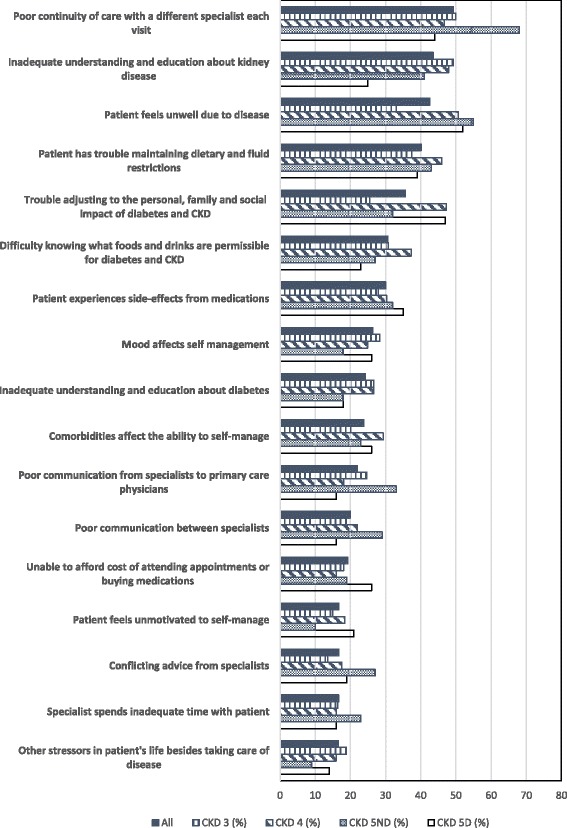
Significant barriers to health-care identified by patients

The commonest barriers to health-care reported by patients (Fig. 3 and Additional file 1: Appendix: Table S3) included poor continuity of care (seeing a different specialist each visit (49.3%)), inadequate understanding and education about CKD (43.5%), feeling unwell (42.6%), and having trouble maintaining dietary and fluid restrictions (40.1%). Other barriers according to CKD stage are shown in the Additional file 1: Appendix: Figure S1: Other barriers to health-care identified by patients.

The significance of each barrier ranked differently across the CKD stages (Fig. 3). Poor continuity of care was the most reported barrier in CKD 5ND (68.2%) and CKD 3 (50.0%) but was the third most reported barrier in CKD 5D (43.9%) and the fourth most reported barrier in CKD 4 (46.7%). Inadequate understanding and education about kidney disease was the second most reported barrier in CKD 3 (49.3%) and CKD 4 (48.0%) and the fourth most reported barrier in CKD 5ND (40.9%). Feeling unwell was the most reported barrier in CKD 4 (50.7%) and CKD 5D (51.8%) but was the second most reported barrier in CKD 5ND (54.6%) and the fourth most reported barrier in CKD 3 (33.1%). Trouble maintaining dietary and fluid restrictions was the third most reported barrier in CKD 3 (37.4%) and 5ND (42.9%) and the fourth most reported barrier in CKD 5D (38.6%). Finally, trouble adjusting to the personal, family and social impact of diabetes and CKD was the second most reported barrier in CKD 5D (47.4%) and the third most reported barrier in CKD 4 (47.3%).

The proportion of patients reporting a particular barrier as a problem also differed across hospitals (Additional file 1: Appendix: Table S3: Barriers to health-care identified by patients per hospital). Across the hospitals, the reported barriers which differed included poor continuity of care due to seeing a different specialist, poor communication from specialists to primary care physicians, poor communication between specialists, the specialist providing inadequate information about the disease and poor relationship with specialist health service staff (Bonferroni *p* < 0.0125). The most reported barrier in hospitals 1 and 3 was poor continuity of care due to seeing a different specialist while the most reported barrier in hospitals 2 and 4 was trouble adjusting to the impact diabetes and CKD has on his/her life and/or that of his/her family and friends (Additional file 1: Appendix: Table S4: The four most common barriers to health-care identified by patients according to hospital).

### Association between recommended care and patient-reported barriers to health-care

There was an association between the following treatment recommendations − an HbA1c of < 8% (64 mmol/mol), receiving an annual eye check in the presence of diabetic retinopathy, and a haemoglobin of 100 – 115 g/L whilst on an ESA − and patient-reported barriers to health-care. Patients who did not have a HbA1c of < 8% (64 mmol/mol) more often reported conflicting advice as an issue (22.4% vs 12.6%, *p* = 0.04), inadequate support from family (15.9% vs 3.2%, *p* < 0.01) and inadequate support from friends (18.2% vs 8.0%, *p* = 0.02) than those who did have a HbA1c of < 8% (64 mmol/mol). Patients who did not receive an annual eye check more often reported poor communication between specialists (57.1% vs 13.3%, *p* = 0.01) and comorbidities affecting the ability to self-manage (66.7% vs 18.9% *p* = 0.02) than those who did receive an annual eye check. Finally, patients on an ESA who did not have a haemoglobin of 100 – 115 g/L more often reported feeling unmotivated to self-manage (29.6% vs 5.3%, *p* = 0.047) than those that did have a haemoglobin of 100 – 115 g/L.

There was no association between the following treatment recommendations − blood pressure < 140/90 mmHg, receiving treatment with a statin if aged > 40 with non-dialysis dependent CKD, having feet examined in the setting of a diagnosis of peripheral neuropathy, and appropriate metformin and ESA prescription − and patient-reported barriers to health-care.

## Discussion

This multi-centre study informs improvement of current models of care for those with co-morbid diabetes and CKD by documenting the presence of gaps between recommended and received care in the tertiary health-care setting and quantitatively identifying the barriers to health-care reported by patients. These barriers varied between hospital and CKD stage, with the most reported barriers being poor continuity of care due to seeing a different specialist, inadequate understanding and education about CKD, feeling unwell and trouble maintaining dietary and fluid restrictions, with some variation according to CKD stage and hospital. Interestingly, these were not associated with recommended care. The only reported barriers associated with a failure to receive recommended care were conflicting advice from specialists and inadequate support from family and friends being associated with a HbA1c ≥ 8% (64 mmol/mol), poor communication between specialists and the effect of co-morbidities on self-management being associated with not receiving an annual eye check in the presence of diabetic retinopathy, and feeling unmotivated to self-manage being associated with a failure to reach a haemoglobin target of 100 – 115 g/L whilst on an ESA.

Our Australian data add to the growing international data demonstrating the presence of guideline-care gaps amongst patients with diabetes and CKD. Despite the lack of information for whom targets may have been individualised (for safety/tolerability reasons), our results suggest significant care gaps with more than 30% of patients having an HbA1c ≥ 8% (64 mmol/mol) and close to 40% of patients having a blood pressure ≥ 140/90 mmHg. The magnitude of these gaps is comparable to other cross-sectional surveys of patients with diabetes and CKD [11, 12]. They are of particular concern given the increased morbidity, mortality and health care costs associated with these conditions. This emphasises the need for strategies to address these gaps in order to build on areas where care is better, such as prescription of statins in non-dialysis patients or annual eye reviews.

The four most reported barriers to health-care were poor continuity of care due to seeing a different specialist, inadequate understanding and education about CKD, feeling unwell and trouble maintaining dietary and fluid restrictions, with some variation according to CKD stage and hospital. Poor continuity of care was mainly reported in hospitals where patients attended clinics with multiple specialists (hospitals 1 and 3), rather than patients attending a clinic where they saw the same clinician. Previous studies amongst patients with CKD alone, have reported that self-awareness of the diagnosis of CKD 3 and 4 was poor [22], and knowledge about CKD and its complications was low [23]. Similarly, patients with CKD have been described to have a high symptom burden, with fatigue, drowsiness, pain and decreased appetite reported in up to 100%, 82%, 90% and 83% of patients with CKD 4 and 5, respectively [24]. Additionally, fluid and dietary restrictions have been recognised to be burdensome and disorienting for patients with CKD with possible strategies to overcome them including patient-prioritised education strategies, using the motivation of avoiding dialysis and viewing adjustment to restrictions as a team effort and journey for patients, their families and their health professionals [25]. Our data suggests that addressing these barriers with patients seeing a single specialist rather than a group of specialists, targeted education of patients about CKD, and individualised symptom control, emotional support and psychological screening and treatment may be very important to patients with co-morbid diabetes and CKD.

Some patient-reported barriers were associated with a failure to receive recommended care. Notably, these barriers were different for different items of recommended care, that is conflicting advice from specialists and inadequate support from family and friends being associated with a HbA1c ≥ 8% (64 mmol/mol); poor communication between specialists and the effect of co-morbidities on self-management was associated with not receiving an annual eye check in the presence of diabetic retinopathy; and feeling unmotivated to self-manage being associated with a failure to reach a haemoglobin target of 100 – 115 g/L whilst on an ESA. This novel finding implies that in order to close the gap between recommended and received care in co-morbid diabetes and CKD, a “one intervention that fits all” approach is unlikely to be successful and that specific interventions aiming to improve each item of recommended care may be required.

Interestingly, the most commonly reported barriers were not necessarily associated with care gaps. There are a few possible explanations for this. Firstly, the achievement of treatment targets and standards of care is an extremely complex interaction between patients and their health-care providers. There are possible health-care provider factors such as a lack of knowledge about treatment targets and standards of care, or treatment inertia [26, 27] which need to be accounted for, and may not be considered by patients. Secondly, the perceptions of different barriers and treatment priorities of health professionals, which are usually delivering recommended care according to guidelines, and of patients may be vastly different. This is consistent with a previous study amongst patients with type 2 diabetes and their health professionals concluding that patients were less aware than physicians concerning many of the barriers to good blood glucose control, high-lighting the need to raise patients’ awareness [28]. Combined with our findings in patients with co-morbid diabetes and CKD, this emphasises the importance of health care systems addressing the key barriers identified by health professionals and also patient-reported barriers known to be associated with suboptimal treatment targets.

Conflicting advice from specialists is a pitfall associated with poor continuity of care and in our study was associated with suboptimal glycaemic control. The exact mechanism behind the association between continuity of care and glycaemic control is ill-defined, although a common denominator in studies of patients with diabetes alone, seems to be shared health information which would most likely lead to consistent advice given by the health professional to the patient [29–32]. This may explain why differing advice from specialists was associated with the level of HbA1c. Similarly, inadequate support from family and friends was also associated with suboptimal glycaemic control. Although not previously reported in co-morbid diabetes and CKD, this is consistent with previous studies amongst patients with diabetes demonstrating a relationship between family and friend support and glycaemic control [33], as well as better self-care [34, 35] and diabetes management [36].

The strengths of this study are the multi-site recruitment of patients from geographically distinct large metropolitan areas and exploration of barriers to health-care identified from previous qualitative work, ensuring that the work is inductive instead of deductive. The study limitations include the inability to make definitive causal inferences because of the cross-sectional study design as well as our inability to consider the impact of individualisation of patient care, especially in the situations where therapeutic goals are appropriately different from the treatment targets. Additionally, we were unable to adequately recruit all ethnic groups or Aboriginal and/or Torres Strait Islander people and exclude responder/selection bias. However, patients (at one tertiary health service) who participated in the study were similar in age, gender distribution and KDOQI CKD stage to those who did not.

## Conclusions

In conclusion, gaps between recommended and received care exist in the management of co-morbid diabetes and CKD in tertiary health-care. Barriers to health-care varied across CKD stage and hospitals. However, barriers related to poor continuity of care were commonly reported by patients and also associated with a failure to reach good glycaemic control. Other barriers associated with a deviation from recommended care were different for different items of recommended care, suggesting that specific interventions aiming to improve each item of recommended care are required to improve health-care for co-morbid diabetes and CKD.

## Additional file

Additional file 1: Supplementary Appendix which contains: Part A: Further information about the Survey Instrument. Part B: Barriers to Health-care Questionnaire. **Table S1.** Characteristics of patients who did and did not participate in the study at one hospital site. **Table S2.** Characteristics and medication usage of patients with diabetes and CKD stratified by CKD stage. **Figure S1.** Other barriers to health-care identified by patients. **Table S3.** Barriers to health-care identified by patients per hospital. **Table S4.** The four most common barriers to health-care identified by patients according to hospital. (DOCX 76 kb)

## Abbreviations

CKD: Chronic kidney disease
GP: General practitioner
HbA1c: Glycated haemoglobin
