# Supplementary material for: Gaps and barriers in health-care provision for co-morbid diabetes and chronic kidney disease: a cross-sectional study
Source: BMC Nephrol. 2017 Feb 28;18:80. doi: 10.1186/s12882-017-0493-x (PMC5331625; doi:10.1186/s12882-017-0493-x)
Supplement: Additional file 1: — Supplementary Appendix which contains: Part A: Further information about the Survey Instrument. Part B: Barriers to Health-care Questionnaire. Table S1. Characteristics of patients who did and did not participate in the study at one hospital site. Table S2. Characteristics and medication usage of patients with diabetes and CKD stratified by CKD stage. Figure S1. Other barriers to health-care identified by patients. Table S3. Barriers to health-care identified by patients per hospital. Table S4. The four most common barriers to health-care identified by patients according to hospital. (DOCX 76 kb) [file 12882_2017_493_MOESM1_ESM.docx]

**Supplementary Appendix**

**Part A: Further information about the Survey Instrument**

Clinical characteristics studied in the first questionnaire included age, gender, language spoken at home, country of birth, body weight, height, body mass index (BMI), systolic and diastolic blood pressure (BP), smoking status, duration of diabetes, type of diabetes, KDIGO (Kidney Disease Improving Global Outcomes) stage [1], dialysis status, presence of hypertension, dyslipidaemia, ischemic heart disease, stroke, peripheral vascular disease, diabetic retinopathy, diabetic neuropathy, diabetic nephropathy as a cause of CKD, biochemical parameters (haemoglobin, HbA1c, total cholesterol, LDL, triglycerides, potassium, calcium, phosphate and PTH), and medication usage (insulin and non-insulin glucose lowering agents; statins, fibrates, ACE inhibitors or Angiotensin 2 Receptor Blockers (ACEIs or AT2RBs), use of other anti-hypertensives and Erythropoeitin stimulating agent (ESA) use).

The second questionnaire completed by the patient consisted of questions examining barriers to health-care (Supplementary Appendix Part B). The questions about barriers to health-care were derived from key themes emerging from the content analysis of qualitative data from 12 focus groups including 58 patients (at each of the 4 hospital sites, separate focus groups were performed for patients with KDOQI (Kidney Disease Outcomes Quality Initiative) CKD stages (3, 4 and 5 [inclusive of 5D]) [2] and 8 semi-structured interviews of carers of patients [3].

**Part B: Barriers to Health-care Questionnaire**

Sometimes people have difficulty looking after their diabetes and kidney disease due to a variety of barriers or obstacles. Listed below are several barriers that may influence your ability to look after your diabetes and kidney disease. To what extent do you agree or disagree with the following factors being a current barrier for you? (Please tick the appropriate box in the table below)

|  | Disagree | Somewhat Disagree | Somewhat Agree | Agree |
| --- | --- | --- | --- | --- |
| My diabetes and kidney specialist does not spend enough time with me | □ | □ | □ | □ |
| My diabetes and kidney specialist does not provide me with enough information/education about my diabetes and kidney disease | □ | □ | □ | □ |
| I am often seen by a different doctor each time I attend my diabetes or kidney disease appointment. | □ | □ | □ | □ |
| My specialists give me conflicting advice | □ | □ | □ | □ |
| I do not have a good relationship with my specialist or other specialist health service staff. | □ | □ | □ | □ |
| Specialist health service staff are not caring, polite and helpful | □ | □ | □ | □ |
| My specialists do not communicate well with my GP | □ | □ | □ | □ |
| My specialists don’t communicate well with each other | □ | □ | □ | □ |
| I do not have a good GP | □ | □ | □ | □ |
| I need more education and understanding of my diabetes | □ | □ | □ | □ |
| I need more education and understanding of my kidney disease | □ | □ | □ | □ |
| The information provided by my doctors or health professionals is hard to understand because English is not my first language or the information is not culturally relevant | □ | □ | □ | □ |
| The information provided by my doctors or health professionals is too complicated | □ | □ | □ | □ |
| It is difficult to obtain medical support and advice for my diabetes when I need it | □ | □ | □ | □ |
| It is difficult to obtain medical support and advice for my kidney disease when I need it | □ | □ | □ | □ |
| I have had an unsatisfactory prior experience with a diabetes or kidney health service/specialist | □ | □ | □ | □ |
| I am unable to afford the cost of attending appointments or buying medication for my diabetes and kidney disease | □ | □ | □ | □ |
| I have trouble adjusting to the impact that diabetes and kidney disease has made on my life and/or that of my family and friends | □ | □ | □ | □ |
| My diabetes and kidney disease makes me feel very unwell | □ | □ | □ | □ |
| My other illnesses affect my ability to look after my diabetes and kidney disease | □ | □ | □ | □ |
| I have many other stressors in my life, and taking care of my diabetes and kidney disease is not a high priority | □ | □ | □ | □ |
| My job makes it difficult to take care of my diabetes and kidney disease well. | □ | □ | □ | □ |
| My mood (e.g. feeling down, worried, frustrated) gets in the way of me looking after my diabetes and kidney disease | □ | □ | □ | □ |
| I do not feel motivated enough to look after my diabetes and kidney disease well | □ | □ | □ | □ |
| I have trouble maintaining the right diet or fluid restriction for my diabetes and kidney disease | □ | □ | □ | □ |
| I have difficulty knowing what I can eat/drink, for my diabetes and kidney disease | □ | □ | □ | □ |
| I experience unpleasant side-effects from my medication | □ | □ | □ | □ |
| I do not receive support from my family | □ | □ | □ | □ |
| I do not receive support from my friends | □ | □ | □ | □ |
| I find it difficult to get services for home-help | □ | □ | □ | □ |

Do you have difficulty accessing a diabetes service?

□ Yes □ No

Do you have difficulty accessing a kidney service?

□ Yes □ No

**Table S1: Characteristics of patients who did and did not participate in the study at one hospital site**

|  | **Participants n = 127** | **Non-participants n = 243** | **P** |
| --- | --- | --- | --- |
| **Age** | 66.6 (10.8) | 68.9 (11.9) | 0.06 |
| **Gender (Male %)** | 69.3 | 60.5 | 0.10 |
| **CKD stage (KDOQI %)** |  |  |  |
| **3** | 34.2 | 40.9 |  |
| **4** | 25.2 | 25.5 |  |
| **5** | 33.9 | 40.3 | 0.37 |

**Table S2: Characteristics and medication usage of patients with diabetes and CKD stratified by CKD stage**

| **Clinical Characteristic** | **All** | **CKD 3** | **CKD 4** | **CKD 5ND** | **CKD 5D** | **P** |
| --- | --- | --- | --- | --- | --- | --- |
| **n (%)** | 308 (100) | 151 (49.0) | 76 (24.7) | 22 (7.1) | 59 (19.2) |  |
| **Age (SD) years** | 66.9 (11.0) | 68.5 (9.4) | 68.1 (12.7) | 62.9 (11.1) | 63.3 (10.6) | 0.002 |
| **Male/Female (%)** | 214 (69.5)/94 (30.5) | 108 (71.5)/43 (28.5) | 51 (67.1)/25 (32.9) | 14 (67.1)/8(32.9) | 41 (69.5)/18 (30.5) | 0.97 |
| **Main language spoken at home (% English)** | 238/305 (78.0) | 114/149 (76.5) | 62/76 (81.6) | 18/22 (81.8) | 44/58 (75.9) | 1.00 |
| **Country of birth Australia** | 141/304 (46.4) | 61/148 (41.2) | 46/76 (60.5) | 12/21 (57.1) | 22/59 (37.3) | 0.45 |
| **Body weight (IQR) kg** | 86.1 (72.8 - 101.3) | 87.8 (75.5 - 105.4) | 89.4 (75.9 - 102.5) | 79.8 (70.6 - 100.5) | 80.1 (62.7 - 94.6) | 0.01 |
| **Height (SD) m** | 1.68 (0.10) | 1.70 (0.10) | 1.69 (0.11) | 1.67 (0.07) | 1.68 (0.10) | 0.81 |
| **BMI (SD) kg/m^2^** | 30.7 (7.4) | 32.3 (6.8) | 31.6 (9.1) | 28.4 (6.1) | 28.2 (5.8) | 0.02 |
| **Systolic blood pressure (SD) mmHg** | 134 (18) | 132 (17) | 134 (18) | 137 (17) | 137 (20) | 0.23 |
| **Diastolic blood pressure (SD) mmHg** | 72 (11) | 71 (11) | 73 (11) | 73 (8) | 73 (13) | 0.32 |
| **Active Smoker (%)** | 18/230 (7.8) | 9/110 (8.2) | 2/54 (3.7) | 6/17 (35.3) | 1/49 (2.0) | 0.001 |
| **Diabetes duration (IQR) yrs** | 17.0 (10.0 -23.5) | 17.9 (10.0 - 23.0) | 19.0 (8.7 - 25.0) | 11.0 (8.0 - 19.0) | 16.0 (12.0-27.0) | 0.21 |
| **Diabetes type (%)** |  |  |  |  |  |  |
| **type 1** | 28 (9.1) | 8 (5.3) | 9 (11.8) | 3 (13.6) | 8 (13.6) | 0.70 |
| **type 2** | 271 (88.0) | 138 (91.4) | 64 (84.2) | 18 (81.8) | 51 (86.4) | 0.81 |
| **other** | 9 (2.9) | 5 (3.3) | 3 (4.0) | 1 (4.6) | 0 (0.0) | 0.41 |
| **Dialysis status (%)** |  |  |  |  |  |  |
| **Non-dialysis** | 249 (80.8) | 151 (100.0) | 76 (100.0) | 22 (100.0) | 0 (0.0) | <0.0001 |
| **Haemodialysis** | 42 (13.6) | 0 (0.0) | 0 (0.0) | 0 (0.0) | 42 (71.2) | <0.0001 |
| **Peritoneal dialysis** | 17 (5.5) | 0 (0.0) | 0 (0.0) | 0 (0.0) | 17 (28.8) | < 0.0001 |
| **Hypertension (%)** | 291 (94.5) | 145 (96.0) | 71 (93.4) | 22 (100.0) | 53 (89.8) | 0.81 |
| **Dyslipidemia (%)** | 256 (83.1) | 128 (84.8) | 57 (75.0) | 21 (95.5) | 50 (84.8) | 0.60 |
| **Ischemic Heart Disease (%)** | 143/304 (47.0) | 71/150 (47.3) | 39/75 (52.0) | 7/21 (33.3) | 26/58 (44.8) | 0.88 |
| **Stroke (%)** | 38/305 (12.5) | 16/150 (10.7) | 14/75 (18.7) | 0/21 (0.0) | 8/59 (13.6) | 0.82 |
| **Peripheral Vascular Disease (%)** | 82/304 (27.0) | 35/150 (23.3) | 24/75 (32.0) | 5/21 (23.8) | 18/58 (31.0) | 0.87 |
| **Diabetic Retinopathy (%)** | 132/305 (43.3) | 57/150 (38.0) | 32/75 (42.7) | 11/22 (50.0) | 32/58 (55.2) | 0.66 |
| **Diabetic Neuropathy (%)** | 108/305 (35.4) | 49/150 (32.7) | 25/75 (33.3) | 5/22 (22.7) | 29/58 (50.0) | 0.52 |
| **Diabetic Nephropathy as a cause of CKD (%)** | 219/306 (71.6) | 104/150 (69.3) | 56/75 (74.7) | 16/22 (72.7) | 43/59 (72.9) | 0.97 |
| **Haemoglobin (SD) g/L** | 122 (18) | 128 (18) | 120 (15) | 115 (11) | 113 (17) | <0.0001 |
| **HbA1c (IQR) (mmol/mol)** | 56 (47 - 67) | 58 (48 - 68) | 57 (48 - 65) | 50 (45 - 61) | 53 (41 - 63) | 0.04 |
| **Total Cholesterol (IQR) mmol/L** | 3.9 (3.4 - 4.6) | 3.8 (3.4 - 4.7) | 3.9 (3.6 - 4.7) | 4.1 (3.6 - 4.7) | 3.8 (3.2 - 4.3) | 0.51 |
| **LDL (IQR) mmol/L** | 1.8 (1.4 - 2.5) | 1.8 (1.4 - 2.6) | 2.0 (1.7 - 2.5) | 2.7 (1.8 - 2.9) | 1.8 ( 1.4 - 2.3) | 0.22 |
| **HDL (IQR) mmol/L** | 1 (0.8 - 1.2) | 1.1 (0.9 - 1.3) | 0.9 (0.7 - 1.1) | 1.2 (1.0 - 1.4) | 1.0 (0.8 - 1.2) | 0.04 |
| **Triglycerides (IQR) mmol/L** | 1.8 (1.2 - 2.5) | 1.7 (1.2 - 2.4) | 2.2 (1.4 - 2.7) | 1.8 (1.1 - 2.5) | 1.7 ((1.3 - 2.3) | 0.27 |
| **Potassium (SD) mmol/L** | 4.6 (0.6) | 4.5 (0.5) | 4.6 (0.6) | 4.6 (0.6) | 4.9 (0.8) | 0.02 |
| **Calcium (SD) mmol/L** | 2.31 (0.14) | 2.36 (0.13) | 2.30 (0.14) | 2.23 (0.13) | 2.27 (0.14) | 0.0001 |
| **Phosphate (IQR) mmol/L** | 1.23 (1.08 - 1.46) | 1.13 (1.03 - 1.23) | 1.30 (1.10 - 1.40) | 1.76 (1.46 - 1.90) | 1.48 (1.26 - 1.96) | 0.0001 |
| **PTH (IQR) pmol/L** | 15.7 (7.8 - 30.6) | 7.7 (4.9 - 11.2) | 15.4 (10.3 - 24.9) | 35.1 (24.3 -44.6) | 35.6 (16.5 - 105.0) | 0.0001 |
| **Treatment of Diabetes (%)** |  |  |  |  |  |  |
| **Insulin only** | 136 (44.2) | 52 (34.4) | 39 (51.3) | 9 (40.9) | 36 (61.0) | 0.28 |
| **Non-insulin glucose lowering therapy only** | 85 (27.6) | 44 (29.1) | 22 (29.0) | 7 (31.8) | 12 (20.3) | 0.91 |
| **Both insulin and non-insulin glucose lowering therapy** | 67 (21.8) | 49 (32.5) | 11 (14.5) | 3 (13.6) | 4 (6.8) | <0.0001 |
| **Diet only without other glucose lowering therapies** | 20 (6.5) | 6 (4.0) | 4 (5.3) | 3 (13.6) | 7 (11.9) | 0.08 |
| **Statin use (%)** | 248 (80.5) | 125 (82.8) | 60 (79.0) | 20 (90.9) | 43 (72.9) | 0.76 |
| **Fibrate (%)** | 34 (11.0) | 20 (13.3) | 8 (10.5) | 1 (4.6) | 5 (8.5) | 0.90 |
| **Treatment of Hypertension** |  |  |  |  |  |  |
| **Use of any antihypertensive (%)** | 278 (90.3) | 140 (92.7) | 72 (94.7) | 22 (100.0) | 44 (74.6) | <0.0001 |
| **Use of ACEI and/or AT2RB (%)** | 185 (60.1) | 111 (73.5) | 46 (60.5) | 8 (36.4) | 20 (33.9) | 0.03 |
| **Use of other antihypertensive besides an ACEI/AT2RB (%)** | 240 (77.9) | 113 (74.8) | 67 (88.2) | 22 (100.0) | 38 (64.4) | 0.28 |
| **ESA use (%)** | 64 (20.8) | 7 (4.6) | 13 (17.1) | 9 (40.9) | 35 (59.3) | 0.0001 |
| **Iron supplementation (%)** | 57 (18.5) | 22 (14.6) | 8 (10.5) | 3 (13.6) | 24 (40.7) | 0.09 |
| **Phosphate binder (%)** | 58 (18.8) | 9 (6.0) | 7 (9.2) | 9 (40.9) | 33 (55.9) | 0.0001 |

ACEI = ACE inhibitor. AT2RB = Angiotensin 2 Receptor Blocker, ESA = Erythopoietin Stimulating Agent

**Figure S1: Other barriers to health-care identified by patients**

**Table S3: Barriers to health-care identified by patients per hospital**

| **Barriers** | **All (%)** | **Hospitals** | | | | |
| --- | --- | --- | --- | --- | --- | --- |
|  |  | **1 (%)** | **2 (%)** | **3 (%)** | **4 (%)** | **P*** |
| **Poor continuity of care with a different specialist each visit** | 147/298 (49.3) | 44/73 (60.3) | 3/35 (8.6) | 91/123 (74.0) | 9/67 (13.4) | < 0.001 |
| **Inadequate understanding and education about kidney disease** | 128/293 (43.5) | 34/73 (46.6) | 11/35 (31.4) | 59/121 (48.8) | 24/65 (36.9) | 0.18 |
| **Patient feels unwell due to disease** | 127/298 (42.6) | 30/73 (41.1) | 11/35 (31.4) | 63/123 (51.2) | 23/67 (34.3) | 0.06 |
| **Patient has trouble maintaining dietary and fluid restrictions** | 120/299 (40.1) | 30/72 (41.7) | 12/36 (33.3) | 53/123 (43.1) | 25/68 (36.8) | 0.68 |
| **Patient has trouble adjusting to the impact diabetes and CKD has on his/her life and/or that of his/her family and friends** | 106/298 (35.6) | 23/73 (31.5) | 13/35 (37.1) | 44/122 (36.1) | 26/68 (38.2) | 0.85 |
| **Patient has difficulty knowing what he/she is allowed to eat and drink for diabetes and CKD** | 93/303 (30.7) | 23/73 (31.5) | 12/36 (33.3) | 40/125 (32.0) | 18/69 (26.1) | 0.82 |
| **Patient experiences side-effects from medications** | 91/302 (30.1) | 24/73 (32.9) | 8/37 (21.6) | 43/124 (34.7) | 16/68 (23.5) | 0.25 |
| **Patient's mood affects self-management** | 80/303 (26.4) | 17/73 (23.3) | 7/37 (18.9) | 41/125 (32.8) | 15/68 (22.1) | 0.19 |
| **Inadequate understanding and education about diabetes** | 73/300 (24.3) | 21/73 (28.8) | 7/35 (20.0) | 28/123 (22.8) | 17/69 (24.6) | 0.73 |
| **Comorbidities affect the ability to self-manage** | 71/298 (23.8) | 21/73 (28.8) | 6/35 (17.1) | 31/123 (25.2) | 13/67 (19.4) | 0.44 |
| **Poor communication from specialists to primary care physicians** | 64/292 (21.9) | 18/72 (25.0) | 3/35 (8.6) | 37/120 (30.8) | 6/65 (9.2) | < 0.01 |
| **Poor communication between specialists** | 56/282 (19.9) | 19/72 (26.4) | 2/34 (5.9) | 29/116 (25.0) | 6/60 (10.0) | < 0.01 |
| **Unable to afford cost of attending appointments or buying medications** | 57/296 (19.3) | 14/72 (19.4) | 6/35 (17.1) | 26/122 (21.3) | 11/67 (16.4) | 0.85 |
| **Specialist spends inadequate time with patient** | 50/300 (16.7) | 18/73 (24.7) | 1/35 (2.9) | 23/124 (18.6) | 8/68 (11.8) | 0.02 |
| **Conflicting advice from specialists** | 49/294 (16.7) | 12/73 (16.4) | 2/35 (5.71) | 28/118 (23.7) | 7/68 (10.3) | 0.03 |
| **Patient feels unmotivated to self-manage** | 50/299 (16.7) | 8/72 (11.11) | 3/36 (8.33) | 25/123 (20.33) | 14/68 (20.6) | 0.16 |
| **Other stressors in patient's life besides taking care of disease** | 50/302 (16.6) | 15/73 (20.6) | 3/36 (8.3) | 24/125 (19.2) | 8/68 (11.8) | 0.24 |
| **Difficulty accessing medical support and advice when required for CKD** | 46/287 (16) | 14/72 (19.4) | 3/34 (8.8) | 20/119 (16.8) | 9/62 (14.5) | 0.58 |
| **Patient has difficulty to get services for home-help** | 43/283 (15.2) | 9/69 (13.0) | 4/33 (12.1) | 23/121 (19.0) | 7/60 (11.7) | 0.55 |
| **Specialist provides inadequate information about the disease** | 42/298 (14.1) | 9/72 (12.5) | 0/34 (0.0) | 26/123 (21.0) | 7/68 (10.3) | < 0.01 |
| **Educational material or information provided is too complicated to understand** | 41/300 (13.7) | 9/73 (12.3) | 8/35 (22.9) | 16/124 (12.9) | 8/68 (11.8) | 0.41 |
| **Unsatisfactory previous experience with a diabetes or kidney health service** | 40/299 (13.4) | 12/73 (16.4) | 3/35 (8.6) | 19/123 (15.5) | 6/68 (8.8) | 0.43 |
| **Trouble accessing kidney service** | 31/283 (11.0) | 6/70 (8.6) | 3/34 (8.8) | 19/123 (15.5) | 3/56 (5.4) | 0.21 |
| **Trouble accessing diabetes service** | 32/297 (10.8) | 7/72 (9.7) | 3/34 (8.8) | 18/125 (14.4) | 4/66 (6.1) | 0.37 |
| **Poor relationship with specialist health service staff** | 31/296 (10.5) | 7/73 (9.6) | 0/35 (0.0) | 21/121 (17.4) | 3/67 (4.5) | < 0.01 |
| **Patient receives inadequate support from his/her friends** | 31/302 (10.3) | 7/72 (9.7) | 1/37 (2.7) | 15/124 (12.1) | 8/69 (11.6) | 0.41 |
| **Difficulty accessing medical support and advice when required for diabetes** | 29/298 (9.7) | 7/72 (9.7) | 3/35 (8.6) | 12/123 (9.8) | 7/68 (10.3) | 1.00 |
| **Educational material or information provided is difficult to understand due to language** | 26/296 (8.8) | 6/73 (8.2) | 4/35 (11.4) | 12/123 (9.8) | 4/65 (6.2) | 0.77 |
| **Patient does not have a good primary physician** | 5/68 (7.4) | 6/72 (8.3) | 2/35 (5.7) | 9/122 (7.4) | 5/68 (7.4) | 0.99 |
| **Patient receives inadequate support from his/her family** | 21/303 (6.9) | 4/73 (5.5) | 0/37 (0.0) | 12/124 (9.7) | 5/69 (7.3) | 0.21 |
| **Specialist health service staff are not caring, polite and helpful** | 18/297 (6.1) | 4/73 (5.5) | 2/35 (5.7) | 10/120 (8.3) | 2/69 (2.9) | 0.54 |
| **Patient's employment makes it difficult to take care of his disease** | 14/271 (5.2) | 2/64 (3.1) | 2/37 (5.4) | 8/107 (7.5) | 2/63 (3.2) | 0.57 |

* p < 0.0125 significant (Bonferroni correction)

**Table S4: The four most common barriers to health-care identified by patients according to hospital**

| **All** | | **Hospital 1** | | **Hospital 2** | | **Hospital 3** | | **Hospital 4** | |
| --- | --- | --- | --- | --- | --- | --- | --- | --- | --- |
| **Barriers** | **N (%)** | **Barriers** | **N (%)** | **Barriers** | **N (%)** | **Barriers** | **N (%)** | **Barriers** | **N (%)** |
| **Poor continuity of care with a different specialist each visit** | 147/298 (49.3) | **Poor continuity of care with a different specialist each visit** | 44/73 (60.3) | **Patient has trouble adjusting to the impact diabetes and CKD has on his/her life and/or that of his/her family and friends** | 13/35 (37.1) | **Poor continuity of care with a different specialist each visit** | 91/123 (74.0) | **Patient has trouble adjusting to the impact diabetes and CKD has on his/her life and/or that of his/her family and friends** | 26/68 (38.2) |
| **Inadequate understanding and education about kidney disease** | 128/294 (43.5) | **Inadequate understanding and education about kidney disease** | 34/73 (46.6) | **Patient has trouble maintaining dietary and fluid restrictions** | 12/36 (33.3) | **Patient feels unwell due to disease** | 63/123 (51.2) | **Inadequate understanding and education about kidney disease** | 24/65 (36.9) |
| **Patient feels unwell due to disease** | 127/298 (42.6) | **Patient has trouble maintaining dietary and fluid restrictions** | 30/72 (41.7) | **Patient has difficulty knowing what he/she is allowed to eat and drink for diabetes and CKD** | 12/36 (33.3) | **Inadequate understanding and education about kidney disease** | 59/121 (48.8) | **Patient has trouble maintaining dietary and fluid restrictions** | 25/68 (36.8) |
| **Patient has trouble maintaining dietary and fluid restrictions** | 120/299 (40.1) | **Patient feels unwell due to disease** | 30/73 (41.1) | **Inadequate understanding and education about kidney disease** | 11/35 (31.4) | **Patient has trouble maintaining dietary and fluid restrictions** | 53/123 (43.1) | **Patient feels unwell due to disease** | 23/67 (34.3) |

**References**

1. Levey AS, de Jong PE, Coresh J, El Nahas M, Astor BC, Matsushita K et al. The definition, classification, and prognosis of chronic kidney disease: a KDIGO Controversies Conference report. Kidney Int. 2011;80(1):17-28. doi:10.1038/ki.2010.483.

2. K/DOQI clinical practice guidelines for chronic kidney disease: evaluation, classification, and stratification. Am J Kidney Dis. 2002;39(2 Suppl 1):S1-266.

3. Lo C, Ilic D, Teede H, Cass A, Fulcher G, Gallagher M et al. The Perspectives of Patients on Health-Care for Co-Morbid Diabetes and Chronic Kidney Disease: A Qualitative Study. PloS one. 2016;Jan 5; 11(1):e0146615.
